# Maresin-1 Alleviates Sepsis-Induced Liver Injury by Regulating Apoptosis and Autophagy via Activation of the PI3K/Akt Signaling Pathway in Mice

**DOI:** 10.3390/cimb48030311

**Published:** 2026-03-13

**Authors:** He Wang, Min Sun, Heng Fan

**Affiliations:** Department of Intensive Care Unit, The First Affiliated Hospital of Ningbo University, Ningbo 315000, China; wanggyf@126.com (H.W.);

**Keywords:** Maresin-1, male mice, autophagy, apoptosis, sepsis-induced liver injury, Akt

## Abstract

Sepsis-induced liver injury (SILI) stands as an independent prognostic factor for mortality among patients diagnosed with sepsis. Maresin-1 (MaR1) is a proresolving lipid mediator. However, its significance in SILI is uncertain. The current study sought to investigate MaR1’s effectiveness in treating SILI, as well as its molecular mechanism. In male C57BL/6J mice, we generated a SILI model by using cecal ligation and puncture (CLP). We further investigated how MaR1 influences inflammation, hepatic autophagy and apoptosis. We showed that treatment with MaR1 ameliorates SILI-induced hepatic injury, as reflected in decreased blood level of the alanine aminotransferase (ALT) and aspartate aminotransferase (AST) enzymes, as well as better appearance of liver tissues. Furthermore, this medication markedly reduced the expression of inflammatory mediators. Importantly, MaR1 inhibited hepatocyte apoptosis by regulating the Bax/Bcl-2 ratio, decreasing cleaved caspase-3 expression, lowering apoptotic cell count, and increasing autophagy. The findings demonstrated that MaR1 treatment reduced p62 protein expression while raising Beclin1 levels and the LC3-II/LC3-I ratio, and facilitated autophagosome formation. The observed effects were most likely due to the stimulation of PI3K/Akt signaling, which was completely prevented by LY294002 (LY), a specific PI3K inhibitor. MaR1’s protective effect in SILI may be mediated via stimulation of the PI3K/Akt pathway, which reduces inflammation and regulates apoptosis and autophagy. Our results give additional experimental evidence of the potential therapeutic uses of MaR1 in the treatment of SILI.

## 1. Introduction

Sepsis, a life-threatening illness, is defined as the dysregulated and exaggerated immunological response to severe infection, which could cause mortality of patients [1]. Nearly 48.9 million sepsis cases occurred globally in 2017, resulting in eleven million deaths and around a fifth of all deaths worldwide [2]. The unique hepatic anatomy and physiology make the organ highly susceptible to microorganisms, inflammatory cytokines, and other factors present in the systemic circulation associated with sepsis [3]. Based on several pieces of research, liver failure is a major indicator of death in sepsis patients, whereas septic hepatitis patients could suffer a mortality rate of up to 40% [4].

MaR1 is a rare specialized pro-resolving mediator that the body produces by manufacturing omega-3 polyunsaturated fatty acid. It has important physiological roles during resolution of inflammation as well as wound healing [5,6]. Studies have shown that MaR1 could inhibit inflammation and promote cell survival, and inhibit apoptosis through different molecular pathways in septic induced organ injury [7,8].

Autophagy is the main pathway of degradation within cells, which contributes to providing nutrients and energy sources needed for cell renewal and homeostasis maintenance [9]. Investigating finds that IL-22 increases autophagy through the ATF4-ATG7 signaling pathway, thus minimizing LPS-induced acute liver damage. These data suggest that autophagy activation is a critical factor in treating sepsis-associated hepatic impairment [10]. The well-known kind of controlled cell death known as apoptosis is typified by nuclear condensation, DNA breakage, and the development of apoptotic bodies. Studies [11,12] have shown that inflammatory cytokines can be secreted during sepsis, inducing liver cell necrosis or apoptosis and ultimately leading to liver damage. There is evidence showing that alpha lipoic acid up-regulates PI3K/Akt signaling, which inhibits apoptosis of hepatocytes and protects against liver injury [13]. Interestingly, recent research on sepsis-induced liver injury has confirmed that MaR1 has multiple liver-protective effects, including anti-inflammatory effects, inhibiting macrophage pyroptosis and ferroptosis [14,15]. Sun et al. reported that MaR1 exerts an anti-AD effect via regulating the activity of ADAM10/17, as well as several downstream protective pathways [16]. The latter also inhibits chronic inflammation and Aβ production through the PI3K/Akt pathway. It can be speculated that MaR1 may protect the liver via regulating the PI3K/Akt pathways.

Against this background, studies investigating the effects of MaR1 on both autophagy and apoptosis in SILI remain limited, particularly with regard to PI3K/Akt pathway, thus constituting a critical research gap. Thus, this study investigated the probable mechanism by which MaR1 controls autophagy and apoptosis through PI3K/Akt signaling to reduce SILI. These findings establish a fresh theoretical paradigm for understanding SILI pathophysiology and create the groundwork for the development of targeted treatment therapies.

## 2. Material and Methods

### 2.1. Animals

The Medical College of Ningbo University supplied male C57BL/6J mice, averaging 20–25 g and aged 6–8 weeks. Mice were kept under SPF living conditions with broad access to food and water, a 12 h light/dark cycle, and a temperature of 23 ± 3 °C. Prior to studies, all mice were acclimated for a period of two weeks. The ARRIVE guidelines for animal studies and all NIH regulations for laboratory animal care were followed in the handling and experimentation of animals. The Ningbo University Institutional Animal Care and Use Committee (No. AEWC2024101, Ningbo, China) approved every procedure.

### 2.2. Animal Model Preparation

Following previous studies [17,18,19], four experimental groups (*n* = 6) were randomly assigned to mice: groups for sham surgery, CLP-induced sepsis, CLP + MaR1, and CLP + MaR1 + LY294002. The intraperitoneal administration of MaR1 (100 ng) proceeded for one hour following surgery. One hour before surgery, LY294002 (0.5 mg/kg) was injected intraperitoneally. The CLP technique was used to generate the sepsis model [20]. Mice were provided with 2% sodium pentobarbital (50 mg/kg, through the peritoneum) to induce anesthesia. To reveal the cecum, a 1.5 cm incision was done in the midline of the abdomen. A 20-gauge needle was used to puncture the distal cecum, and the contents of the feces were then carefully extruded. For fluid resuscitation, 1 mL of sterile saline was inserted subcutaneously after the cecum was replaced and the wound itself was closed in layers. For the sham-surgery control group, the protocol involved rewarming the animal and maintaining its body temperature. The method was the same as for the CLP group, with the exception of a crucial step: caecal puncture and ligation. A total of 24 h after CLP, the animals were sacrificed. Then, 2% sodium pentobarbita was injected intraperitoneally to produce anesthesia prior to euthanasia. Cervical dislocation was used for euthanasia when profound anesthesia was achieved, which usually took three to five minutes.

### 2.3. Reagents

MaR1: Cayman Chemical Inc., product no. 10878, Ann Arbor, MI, USA. LY294002: MedChemExpress, product no. HY-10108, Monmouth Junction, NJ, USA. Primary antibodies used for immunodetection are detailed as follows: GAPDH (ab181602, Abcam, Cambridge, MA, USA); autophagy analysis kit containing LC3, Beclin-1 and p62 antibody (Cat No: 4445, CST, Danvers, MA, USA); Bcl-2 and Bax antibodies (#50599-2-Ig and #26593-1-AP, respectively) from Proteintech (Wuhan, China); cleaved caspase-3 antibody (Catalog No. 9661; CST, Danvers, MA, USA); antibody to phospho-Akt (S473), Akt antibody (Cat No: 9271, Cat No: 9272, CST, Danvers, MA, USA); secondary antibodies, which include anti-mouse and anti-rabbit (from Invitrogen, Carlsbad, CA, USA). TNF–α, IL–6, and IL–1β ELISA kits (cat. nos. 88–7324–86, 88–7064–86 and 88–7013A–88, Pierce Biotechnology, Rockford, IL, USA).

### 2.4. Liver Function and Serum Inflammatory Factor Detection

The commercial detection kits (Cat. No. C009-2-1 and C010-2-1) used for evaluating the levels of ALT and AST in serum were given by the Nanjing Jiancheng Bioengineering Institute in Nanjing, China. Using the following reagents, the activity was measured: 20 μL of sera samples were combined with the reaction solution described above, and using a microplate reader set to 510 nm, the optical density value has been identified. Then, using the normal procedures that came with the kit, the enzyme activity was determined.

Basically, 50 µL of serum sample was put into wells that had already been coated, and they were left to incubate for two hours at room temperature. To get rid of any loose debris, it was then given three TBST washes. An HRP-labeled detection antibody was then added to each well, and the mixture was permitted to sit at room temperature for an hour. Three TBST washes were used to eliminate the free detection antibody. The mixture was incubated for 15 min at room temperature after 50 μL of TMB substrate was applied. We measured the absorbance at 450 nm after introducing 25 μL of H_2_SO_4_ to the solution.

### 2.5. Liver Histology Staining and Evaluation

Before being upheld in paraffin wax, liver samples from the left lobes had been initially dehydrated in alcohol gradients and then fixed with 4% paraformaldehyde. The tissues were stained with H&E and observed under a light microscope (Nikon, Tokyo, Japan) after being divided into 5 μm pieces. Two observers who were blinded to the experimental groups conducted the histopathological analysis: Grades 0–4 for liver injury include: 0, normal hepatic structure; 1, mild hepatic stasis, cytoplasmic vacuolization, or isolated necrotic cells; 2 pathologic change <30% of examined surface; 3 injury covering no more than 60% of the microscope view; and 4 widespread bleeding and necrosis covering more than 60% of the histological section, according to work [21].

### 2.6. Immunohistochemistry Assay

Those paraffin-free elements were accessible via microwave for antigen. Slices were blocked with 2.5% normal goat serum before logging incubated with 1:200 diluted initial antibodies for a whole evening at 4 °C. Afterwards, PBS was utilized to rinse them three times. An Olympus microscope was used to take histology pictures at 400× magnification following a 30 min incubation period with biotinylated secondary antibodies at room temperature. Protein expression was assessed by measuring integrated optical density (IOD) with ImageJ 1.53e software (National Institutes of Health, Bethesda, MD, USA).

### 2.7. TUNEL Assay

Samples from paraffin-fixed liver tissue were sliced into 4 μm slices. Sections were digested by using proteinase K enzyme under 37 °C temperature during 20 min, and then washed thoroughly using PBS. TUNEL staining was implemented using a commercial kit (Roche, Basel, Switzerland) as directed, with 60 min incubation at 37 °C. Subsequent to thorough rinsing with PBS, the sections were counterstained with DAPI, dehydrated through an ethanol gradient, cleared, mounted using antifade medium, and analyzed with a fluorescence microscope (Olympus, Tokyo, Japan). For apoptotic cell counting, six random visual fields of each histological slice were counted for TUNEL positive cells under the objective lens of 400× magnification.

### 2.8. Utilizing Transmission Electron Microscopy for Observing Autophagosomes

2.5% glutaraldehyde and 2% paraformaldehyde in 0.1 M phosphate buffer were utilized to fix the liver tissues for six hours at room temperature, alongside one extra hour at 4 °C. After fixation, 1 h of 1% osmium tetroxide was applied. The samples have been embedded in Epon 812 resin after being rinsed with deionized water. Using a Hitachi H 600 transmission electron microscope (Tokyo, Japan), ultrathin slices of around 70 nm were generated using a LKB V ultramicrotome (LKB Instrument AB, Bromma, Sweden), stained with 2% uranyl acetate and 0.2% lead citrate, and seen (Tokyo, Japan) [22].

### 2.9. Western Blot Analysis

RIPA buffer (P0013B, Beyotime, Shanghai, China) has been utilized to lyse liver tissues with the intent to extract proteins. The protein content has been evaluated using a BCA kit (P0009, Beyotime, Shanghai, China). The same number of protein (30 µg/lane) was transferred to PVDF membranes (Millipore, Burlington, MA, USA) for subsequent splitting by 10% SDS-PAGE. The membranes had been clogged with 5% non-fat milk for an hour at room temperature before being cleaned with TBST. Afterwards, primary antibodies were incubated overnight at 4 °C; membranes were incubated for an hour with HRP-conjugated secondary antibodies (Thermo Fisher Scientific, Waltham, MA, USA, 1:10,000; Proteintech, Wuhan, China, 1:5000). The protein bands have been detected on ECL, acquired using a UVP imaging gadget, and calculated using Image J.

Band intensity quantification: The ImageJ program, version 1.53e, was used for the quantification of bands’ intensities in our study; the following primary antibodies were used: antibodies against GAPDH; antibodies against anti-Bax, LC3, cleaved caspase-3, beclin-1, p62, Bcl-2 and antibodies; antibodies to Phospho-Akt and Akt. The relative expression levels of proteins for Bax, cleaved caspase-3, beclin-1, p62, and Bcl-2 were normalized to GAPDH. The ratio of LC3-II to LC3-I was computed for LC3. The relative phosphorylation levels for Phospho-Akt were normalized to total Akt. Every experiment was carried out in triplicate, and statistical analysis was applied to the data.

### 2.10. Quantitative Real-Time PCR Analysis

Using TRIzol (Invitrogen, Carlsbad, CA, USA), total RNA was extracted from liver and determined by using NanoDrop (Wilmington, DE, USA). the PrimeScript RT Kit (TaKaRa, Kusatsu, Shiga, Japan) to synthesize cDNA. Using SYBR Premix Ex Taq II, qPCR was carried out in 20 μL at 95 °C for 10 min, 40 cycles (95 °C 15 s, 60 °C 30 s), and melting curve analysis for specificity. To measure the expression of the target gene, the 2^−ΔΔCt^ method had been used [23], with β-actin acting as the internal control gene [24]. All of the sequences of TaKaRa’s commercially produced primers used were as follows (Table 1).

### 2.11. Statistical Analysis

Before analysis, the normality of the data was examined by the Shapiro–Wilk test. Every intergroup comparison was performed in this study. Mean ± standard deviation (mean ± SD) was utilized to display data with a normal distribution. Statistical analysis was executed using IBM SPSS 21.0 and GraphPad Prism 5. Tukey’s post hoc test has been used post one-way ANOVA for multiple group comparisons. Statistically significant values were defined as *p* values less than 0.05.

## 3. Results

### 3.1. MaR1 Alleviates Sepsis-Induced Liver Dysfunction After CLP

**Figure 1:**
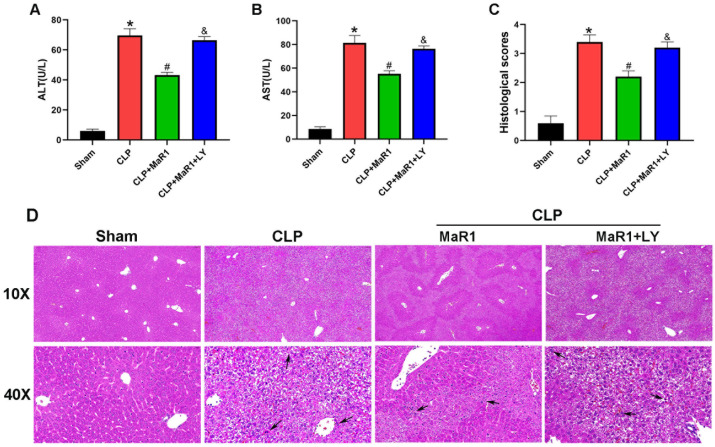
(**A**,**B**) ALT and AST levels in the four groups. MaR1: Maresin-1, LY: LY294002. (**C**) Quantitative evaluation of liver histopathological damage scores. (**D**) Histopathological examination of liver tissues through H&E staining. Black arrows denoted the regions with inflammatory cell infiltration (100× and 400× magnification; bar = 50 μm). * *p* < 0.05 in comparison to the Sham group, # *p* < 0.05 in comparison to the CLP group and & *p* < 0.05 in comparison to the CLP + MaR1 group. The data are shown as mean ± SD (*n* = 6, per group).

In an effort to provide insight into the regulatory role of MaR1 in SILI, we analyzed the serum levels of ALT and AST in each mouse group 24 h after CLP. The CLP group’s serum ALT and AST amounts were substantially higher compared with those of the sham group. The CLP + MaR1 group’s serum ALT and AST levels were much lower compared to those of the CLP group, suggesting that MaR1 treatment effectively reversed the increase in these liver function indexes after CLP. However, when MaR1 and LY294002 were given together, MaR1’s protective effect on serum ALT and AST levels was eliminated (Figure 1A,B). Furthermore, liver histological analysis showed that the CLP group had more severe liver damage following surgery than the sham group. The extent of liver damage was considerably reduced following MaR1 therapy. In particular, the CLP + MaR1 group showed less pathological liver damage than the CLP group. However, the coadministration of MaR1 and LY294002 eliminated the ability of MaR1 to mitigate liver pathological damage (Figure 1C,D). These results collectively demonstrated that MaR1 protects against liver dysfunction induced by CLP-induced sepsis.

### 3.2. MaR1 Ameliorates Inflammatory Responses in Liver Tissue

**Figure 2:**
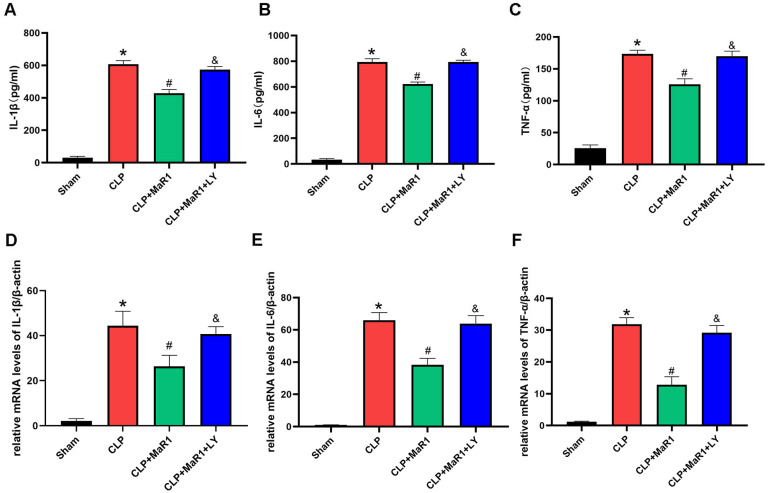
(**A**–**C**) The levels of blood of inflammatory factors TNF-α, IL-1β, and IL-6. MaR1: Maresin-1, LY: LY294002. (**D**–**F**) β-actin was used as an internal reference gene to evaluate and change the relative mRNA expression of Tnf-α, IL-1β, and IL-6. * *p* < 0.05 in comparison to the Sham group, # *p* < 0.05 in comparison to the CLP group and & *p* < 0.05 in comparison to the CLP + MaR1 group. The data are shown as mean ± SD (*n* = 6, per group).

Sepsis exacerbates the systemic infection and promotes overproduction of inflammatory mediators [11]. We measured the inflammatory response after CLP by measuring serum concentrations of TNF-α, IL-1β and IL-6 in each group and then examined the potential connection between inflammatory suppression and MaR1’s inhibitory contribution to sepsis-induced liver damage (Figure 2). The CLP group’s serum levels of TNF-α, IL-6, and IL-1β were significantly greater than those of the sham-operated group. But following CLP, MaR1 treatment reduced these inflammatory factors’ levels: the CLP + MaR1 group had lower blood levels of TNF-α, IL-1β, and IL-6 than the CLP group. However, when MaR1 and LY294002 were taken combined, the beneficial impact of MaR1 on serum IL 1β, IL 6, and TNF α levels was totally reversed (Figure 2A–C). Moreover, the qRT-PCR results aligned with the serum cytokine data (Figure 2–F). Therefore, following CLP, MaR1 inhibited the amounts of inflammatory mediators in hepatic tissue.

### 3.3. MaR1 Enhances Hepatocyte Autophagy During SILI

**Figure 3:**
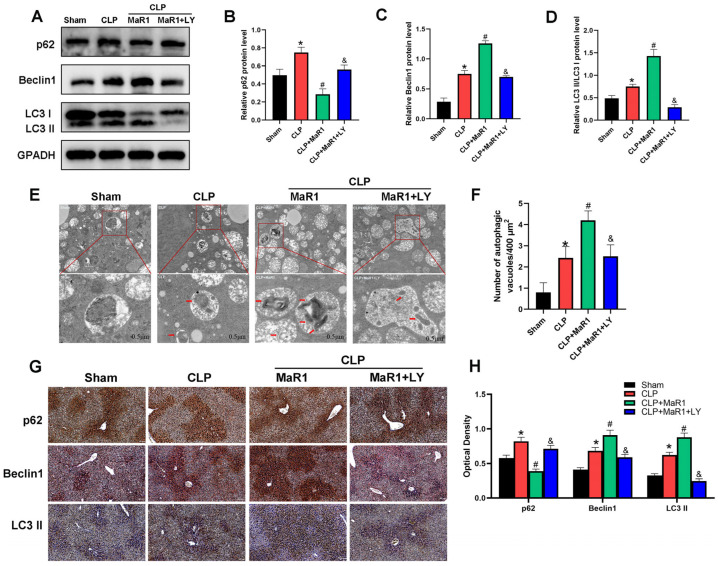
(**A**) Western blot detection of p62, Beclin-1, and LC3 II/I levels. MaR1: Maresin-1, LY: LY294002. (**B**–**D**) Quantitative evaluation of p62, Beclin-1, and LC3 II/I protein expression. (**E**) Representative TEM images of autolysosomes (red arrows) in liver tissues. Magnification, 20,000×. (**F**) Quantitative analysis of autophagosome quantity. (**G**) Representative immunohistochemistry images of liver tissues showing p62, Beclin1, and LC3 II/I (200× magnification; bar = 50 μm). (**H**) Quantitative IHC examination of liver tissue p62, Beclin1, and LC3 II protein expression. * *p* < 0.05 in comparison to the Sham group, # *p* < 0.05 in comparison to the CLP group and & *p* < 0.05 in comparison to the CLP + MaR1 group. The data are shown as mean ± SD (*n* = 6, per group).

The results of previous studies demonstrated that autophagy induction might mitigate the harm that sepsis brings to the liver [25,26,27]. We therefore examined core autophagic protein levels to verify if this pathway mediates the effects of MaR1 on SILI. In comparison to the sham group, mice exposed to CLP showed decreased p62 expression and elevated levels of Beclin1 and LC3-II proteins, suggesting an increase in the expression of protein markers linked to autophagy (Figure 3). MaR1 treatment further enhanced autophagic activity in hepatocytes: Beclin1 and LC3-II protein levels were significantly higher in the CLP + MaR1 group than in the CLP group, while p62 protein expression was further reduced. Interestingly, the coadministration of LY294002 abrogated the regulatory effect of MaR1 on Beclin1, p62, and LC3-II protein expression (Figure 3A–D). In addition, we observed autophagosomes in liver tissue via TEM. The results regarding autophagosome formation were consistent with the protein data: CLP surgery increased autophagosome accumulation compared with the sham group, and MaR1 further promoted this process. However, coadministration of LY294002 eliminated the ability of MaR1 to promote autophagosome formation (Figure 3E,F). Furthermore, the immunohistochemistry results for liver tissue were consistent with those of Western blotting and TEM (Figure 3G,H). All of these results indicate which MaR1 can successfully alter autophagy-related markers in liver tissue during SILI.

### 3.4. MaR1 Inhibits Hepatocyte Apoptosis During SILI

**Figure 4:**
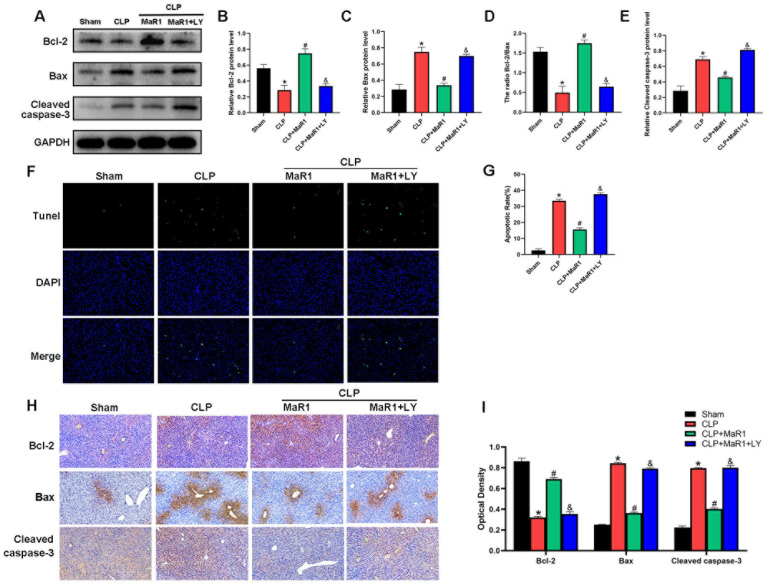
(**A**) Cleaved caspase-3, Bax, and Bcl-2 protein expression levels were evaluated by Western blot analysis. (**B**–**E**) Quantitative assessment of cleaved caspase-3 protein expression levels, Bcl-2, Bax, and the Bcl-2/Bax ratio. MaR1: Maresin-1, LY: LY294002. (**F**) The amount of liver tissue apoptosis in each group; (**G**) the quantity of liver tissue apoptosis. (**H**) Hepatic tissue IHC staining for Bcl-2, Bax, and cleaved caspase 3 (200× magnification; bar = 50 μm). (**I**) Quantitative assessment of cleaved caspase-3, Bax, and Bcl-2 expression in hepatic tissues using IHC. * *p* < 0.05 in comparison to the Sham group, # *p* < 0.05 in comparison to the CLP group and & *p* < 0.05 in comparison to the CLP + MaR1 group. The data are shown as mean ± SD (*n* = 6, per group).

Autophagy and apoptosis often crosstalk in various diseases [28,29,30]. Thus, after confirming that MaR1 enhances cellular autophagy, we looked for proteins linked to apoptosis in order to gauge the extent of hepatocyte death in mice following CLP (Figure 4). The CLP group showed significantly higher levels of Bax and cleaved caspase-3 protein expression and lower levels of Bcl-2 expression in comparison to the sham group. MaR1 injections significantly raised the expression of Bcl-2 protein while considerably reducing the expression of Bax and cleaved caspase-3, in contrast to the CLP group. Interestingly, the effects of MaR1 on Bax, cleaved caspase-3, and Bcl-2 expression were reversed when LY294002 was administered in conjunction with MaR1 (Figure 4A–E). We stained the liver tissues from each group with TUNEL to confirm the degree of hepatocyte apoptosis. The number of apoptotic cells was significantly higher in the CLP group than in the sham group. On the other hand, apoptosis was considerably reduced in the CLP + MaR1 group as compared to the CLP group by MaR1 therapy. However, the anti-apoptotic effect of MaR1 was eliminated when LY294002 was administered concurrently (Figure 4F,G). Additionally, the results of IHC staining of liver tissue were consistent with those of Western blotting and TUNEL staining (Figure 4H,I). Collectively, these findings indicate that MaR1 can effectively inhibit hepatocyte apoptosis during SILI.

### 3.5. MaR1 Activates the PI3K/Akt Signaling Pathway for Regulation of Autophagy and Apoptosis

**Figure 5:**
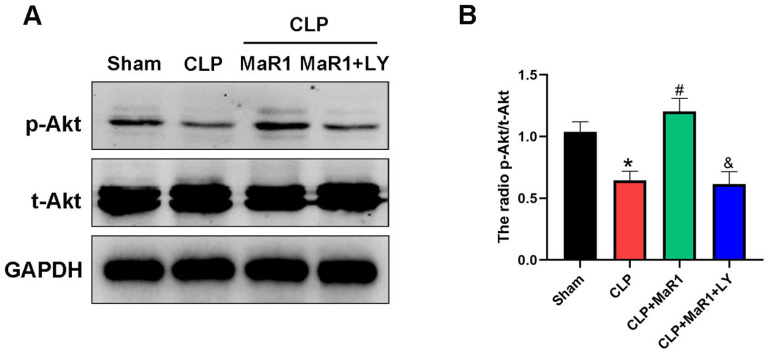
(**A**) Hepatic tissues’ levels of the p-Akt and t-Akt. MaR1: Maresin-1, LY: LY294002. (**B**) Quantification research on total Akt and p-Akt protein expression. * *p* < 0.05 in comparison to the Sham group, # *p* < 0.05 in comparison to the CLP group and & *p* < 0.05 in comparison to the CLP + MaR1 group. The data are shown as mean ± SD (*n* = 6, per group).

Based on Western blot analysis, the CLP group’s liver tissue had a significantly lower p-Akt/t-Akt ratio than the sham group. On the other hand, co-treatment with LY294002 eliminated the impact of MaR1 therapy, which significantly increased p-Akt expression in comparison to the CLP group (Figure 5A,B). Collectively, these results show that MaR1 reduces sepsis liver damage by promoting autophagy and suppressing hepatocyte apoptosis via the PI3K/Akt signaling pathway.

## 4. Discussion

**Figure 6:**
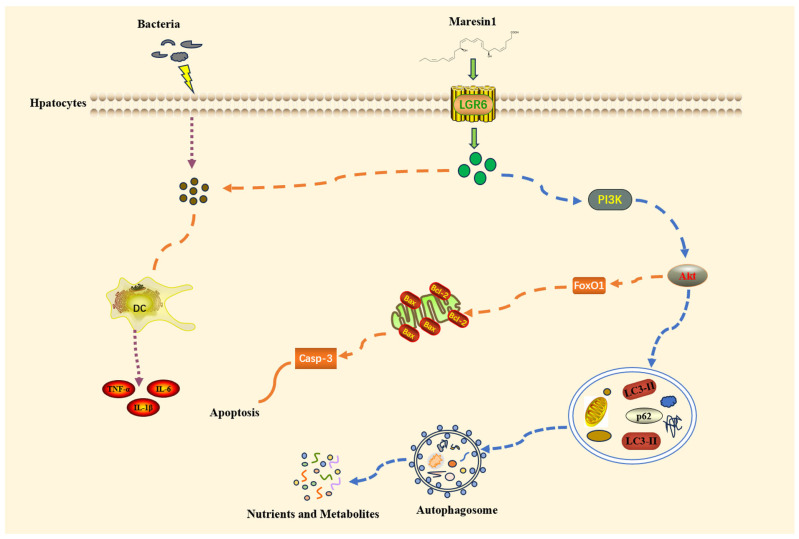
Diagrammatic representation of how MaR1 could utilize the PI3K/Akt pathway to mediate autophagy and apoptosis.

MaR1 is a unique kind of physiologically active lipid that fosters wound healing and has potent anti-inflammatory properties. It has a preventive effect against sepsis and associated organ damage, according to earlier research. [31,32,33]. The study’s findings demonstrated that MaR1 reduces SILI by controlling the PI3K/Akt pathway (Figure 6). It is worth noting that these protective effects could be entirely abolished by using LY294002, a PI3K inhibitor; thus, we provide another way via which MaR1 may exert its function during SILI. This new finding has not been reported before. Our results collectively show that MaR1 may be a good candidate medication for future SILI patient therapy.

Inflammation serves an integral part in the development of sepsis; an overabundance of inflammatory mediators upsets the body’s inflammatory rhythm, which leads to insufficient tissue perfusion and ultimately organ failure [34,35,36]. Autophagy is an intrinsic recycling process at the cell level, where cell constituents, or invading microbes, are contained in intracellular vesicles which are transported towards the lysosome and degraded there. Along with preserving cell homeostasis and promoting cell survival, this mechanism also plays a role in inflammation and immunity [37,38]. It was reported that the MaR1 could be used to treat septic inflammation [39]. Corroborating these data, our study revealed that MaR1 attenuated inflammatory responses in septic mice and thereby exerted a protective effect. Furthermore, we observed that MaR1 may exert its protective role by upregulating autophagy in hepatocytes. However, the detailed mechanism by which it regulates autophagy remains to be verified in the current study.

Autophagy is another phenomenon which has a close relationship to apoptosis. It is well known that autophagy and apoptosis are tightly connected and both of them have crucial roles during stress response in cells. In this regard, it was shown that interaction of Bcl2 and Beclin1 proteins, and through formation of the Beclin1-Vps34 complex, could be key regulators within this process, although it is not clear which precise molecular mechanisms are involved [40]. Importantly, it has recently been shown that inhibition of apoptosis could ameliorate SILI [41]. We utilized Western blot and TUNEL test to measure apoptotic levels after observing that MaR1 controlled the expression of autophagy markers in hepatocytes. The information verified that MaR1 had an anti-apoptotic impact during SILI. Together, our results suggest that MaR1’s hepatoprotective effect may be achieved, at least partially, by suppressing apoptotic cell death and coordinating autophagic activity. Both autophagy and apoptosis are controlled through various signaling pathways, with the PI3K/Akt pathway operating as a vital regulator for both [42]. Hepatic injury caused by sepsis has been shown to be substantially lowered by increasing the PI3K/Akt signaling cascade [43]. Administration of LY294002 to inhibit PI3K/Akt signaling increased serum proinflammatory mediators, suppressed autophagy, and promoted hepatocyte apoptosis, confirming that LY294002 counteracts the role of MaR1 on the liver.

There are a few limitations to this study that should be noted. One major restriction is the absence of numerous MaR1 dosages and time periods (only 24 h after CLP). Second, LY294002 is a non-specific PI3K inhibitor that may have off-target effects, even though we utilized it to investigate how MaR1 modulates autophagy, apoptosis, and the PI3K/Akt pathway in SILI. Thus, more specific inhibitors or agonists targeting the PI3K/Akt cascade are needed to verify the exact regulatory mechanism of MaR1 in SILI. Furthermore, the molecular basis underlying autophagy and apoptosis regulation is extremely complex, and additional studies are required to identify other potential pathways that may contribute to SILI.

## 5. Conclusions

In conclusion, our study proves that MaR1 modulates SILI in mice via modulating autophagy and apoptosis by the PI3K/Akt signaling pathway. We will focus on clarifying the complex molecular mechanism by which MaR1 regulates the PI3K/Akt pathway in order to confirm its potential therapeutic effect for SILI, and we will conduct thorough investigations in future research.

## Figures and Tables

**Table 1:** Primer sequences in reverse transcription-quantitative polymerase chain reaction.

*Gene*	*Forward Sequence (5′ → 3′)*	*Reverse Sequence (5′ → 3′)*
IL-1β	TCGCAGCAGCACATCAACAAGAG	AGGTCCACGGGAAAGACACAGG
IL-6	TCTGGAGCCCACCAAGAACGATAG	GTCACCAGCATCAGTCCCAAGAAG
TNF-α	GGACTAGCCAGGAGGGAGAACAG	GCCAGTGAGTGAAAGGGACAGAAC
β-Actin	CTACCTCATGAAGATCCTGACC	CACAGCTTCTCTTTGATGTCAC

## Data Availability

The original contributions presented in this study are included in the article. Further inquiries can be directed to the corresponding author.
